# First Report of Mitochondrial DNA Copy Number Variation in *Opsius heydeni* (Insecta, Hemiptera, Cicadellidae) from Polluted and Control Sites

**DOI:** 10.3390/ani13111793

**Published:** 2023-05-28

**Authors:** Giada Santa Calogero, Marta Giuga, Vera D’Urso, Venera Ferrito, Anna Maria Pappalardo

**Affiliations:** 1 Department of Biological, Geological and Environmental Sciences, Section of Animal Biology “M. La Greca”, University of Catania, Via Androne 81, 95124 Catania, Italy; giadacalogero@gmail.com (G.S.C.); marta.giuga@phd.unict.it (M.G.); dursove@unict.it (V.D.); 2 Institute for the Study of Anthropic Impact and Sustainability in the Marine Environment-National Research Council, Via del Mare 3, 91021 Trapani, Italy

**Keywords:** *Opsius*, Insecta, mtDNA copy number, real-time PCR, biomarker, pollution

## Abstract

**Simple Summary:**

Industrial activities significantly damage the surrounding environment due to the release of contaminants of various types. Therefore, the search for parameters that can provide information on the quality of the environment is of great relevance. In this research, for the first time, specimens belonging to *Opsius heydeni*, an insect living on *Tamarix* plants, were sampled and analyzed at two Sicilian sites (the polluted industrial site Priolo Gargallo and the control site Oasi del Simeto) to study the effect of pollution on wildlife organisms. Using molecular biology techniques, it has been seen that environmental pollutants can modify the mitochondrial DNA in these specimens; in particular, a decrease in the number of DNA molecules was observed in the specimens sampled at Priolo Gargallo. In conclusion, these results highlight how the studied species can be useful to monitor the effects of pollutants on biota.

**Abstract:**

Mitochondrial DNA easily undergoes alterations due to exposure to stress factors. In particular, mitochondrial DNA copy number (mtDNAcn) variation can be used as a biomarker of the effect of exposure to various environmental contaminants. In this study, a molecular investigation based on the evaluation of mtDNAcn variation was applied for the first time to individuals belonging to the species *Opsius heydeni*. A total of 20 samples were collected from two sites in eastern Sicily: Priolo Gargallo, a site with a strong anthropic impact, and the Simeto river Oasis, a control site. Specimens identified based on morphological traits were used to obtain *COI* gene sequences from this species that were not previously available in GenBank. After processing, the relative mtDNAcn was evaluated using real-time PCR of a portion of the *COI* and *18S* genes. A decrease in the mtDNAcn in the specimens from the polluted site was observed. These results highlight how environmental contaminants can alter the mitochondrial genome and how *Opsius heydeni* can be considered a potential bioindicator species of environmental quality.

## 1. Introduction

During the last century, many Mediterranean areas along the coast were affected by anthropogenic disturbances arising from many industrial activities that released wastes and contaminants belonging to several classes into the environment. Some of them are dramatically toxic and persist in the environment for long periods, generating a process known as biomagnification [1]. Furthermore, persistent contaminants may remain firmly associated with marine sediments for a long time after cessation of active inputs [2] and, as effects of specific hydrological and/or chemical conditions, undergo sudden releases that represent a serious threat to marine biota [3,4]. It is also known that a wide range of environmental contaminants induce the production of reactive oxygen species (ROS) that can cause severe alterations to biological macromolecules such as proteins, lipids, and DNA, so much so that a positive correlation has been established between oxidative damage to nuclear DNA and chemical contaminants [5].

Unlike nuclear DNA (N), mitochondrial DNA (mtDNA) is particularly vulnerable to various types of damage due to the lack of protective histones but especially because it is in the proximity of ROS generation sites, which are located within complex I and III of the electron transport chain [6]. As a result of the extreme sensitivity of the mitochondria to exposure to stressors, the number of copies of the mitochondrial genome does not remain constant over time. Indeed, various studies, conducted in vivo and in vitro, have shown that, when exposed to various genotoxins, the mitochondria undergo damage, including an alteration in the number of copies of the mitochondrial genome itself [7]. In particular, the intracellular increase in ROS leads to an increase in mitochondrial biogenesis as an adaptive response that can be detected by measuring the mt/N ratio. The persistence of oxidative stress can cause damage to mitochondrial membranes, proteins, and DNA over time, causing mitochondrial dysfunction with a consequent long-term decrease in the mt/N ratio. Therefore, it has been hypothesized that an increase or decrease in the mt/N ratio could precede the onset of mitochondrial dysfunction and that mitochondrial DNA damage could be an excellent early biomarker preceding the eventual collapse of mitochondrial function [8]. The mitochondrial DNA copy number (mtDNAcn) has proven to be an easy-to-use biomarker of mitochondrial damage and has been confirmed as an excellent biomarker in various human pathologies such as cardiovascular disease [9], cancer [10], diabetes [11], and many others.

Analysis of the effects generated by toxins and pollutants in a variety of animal species has further demonstrated that exposure to various environmental stress factors has an impact on mtDNAcn. In *Drosophila melanogaster*, for example, an increase in mitochondrial genome copy number was observed following exposure to exogenous agents such as ochratoxin A and methanol [12], but also in rats following passive cigarette smoke exposure both in utero and throughout the neonatal period [13].

When the mitochondrial damage in question is assumed to be caused by exposure to pollutants, it must be considered that it is not specific; in fact, various pollutants can cause alterations in this variable. Scientific research has mostly focused on the use of mtDNAcn variation in the study of human pathologies. Little data is available on the use of mitochondrial DNA as a biomarker of the effects of environmental contaminants on wildlife.

In this context, the highly industrialized area of Augusta (SE Sicily-Italy), which has one of the largest petrochemical plants in Europe, is considered a site of high environmental risk, both on the Italian [14] and international [15] scales. Industrial activities in this area began in the 1950s and developed rapidly until the 1980s; then, some industries were subsequently dismissed, while others are now present. The most worrying pollutant in the sediment is Hg, resulting from a former chlor-alkali plant, to which other metals and organic compounds such as polycyclic aromatic hydrocarbons (PAH), polychlorinated biphenyls (PCBs), and hexachlorobenzene (HCB), discharged mainly from petrochemical industries, have been added over time. As a result, high levels of complexity and diversity of contamination characterize this area, and local populations are continuously exposed to various contaminants through multiple pathways [16,17]. For these reasons, the area was listed as a Site of National Interest (SNI) by the Italian Ministry of the Environment in 2003 and named ‘Priolo Gargallo’, including both a terrestrial and marine area. The latter incorporates the natural harbor of Augusta and a large coastal marine area reaching as far as Syracuse, which extends 3 km offshore [16,17,18]. Within this perimeter, the Magnisi peninsula is heavily contaminated and is the site of a pyrite ash dump resulting from industrialization activities in the surrounding area [19]. Therefore, the peninsula was identified as the study area in this research. The natural reserve “Simeto Oasis”, a protected area established in 1984 at the mouth of the Simeto river, has been chosen as a control site.

*Opsius heydeni* Lethierryi (Insecta, Hemiptera, Cicadellidae), a sap-feeder insect, was chosen as a bioindicator to assess the negative anthropic impact on the polluted site Priolo Gargallo. The species belongs to a genus with at least 20 recognized species found worldwide, overall living in damp areas with *Tamarix* spp. (Tamaricaceae), particularly river valleys [20].

*Tamarix* is a genus in the Tamaricaceae family, which has 78 species primarily adapted to arid and saline environments [21]. *Tamarix* species may be found in a wide range of arid environments, including deserts and semi-deserts, saline sandy soils, saline wetlands, and riverine deltas. They are found in many polluted areas and have been classified as metal accumulators [22,23,24].

*O. heydeni*, distributed from Turan to the Arabian Peninsula up to North Africa, Europe, the Canary Islands, and the Azores, completes the entire life cycle on *Tamarix* spp. [20]. As in our collection sites, *O*. *heydeni* often coexists with the very similar *O. stactogalus* Fieber, 1866, with which it shares the general appearance and dimensions; the two species can easily be distinguished from the examination of the male genital apparatus. For this reason, it was decided to use only male specimens to be sure of the specific morphologically based identification. Therefore, based on the previous considerations, the aim of this study is to extend the use of mtDNAcn variation to determine the effects of environmental pollutants on populations of a selected species of invertebrates in polluted and control environments.

## 2. Materials and Methods

### 2.1. Sampling

**Figure 1:**
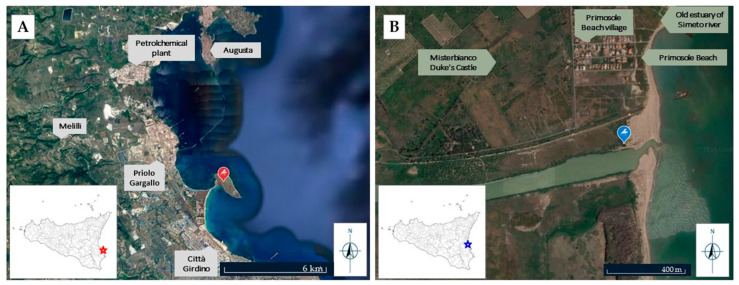
(**A**) Polluted sampling site on the Magnisi peninsula (37°09′15′′ N, 15°13′56′′ E), red placeholder. (**B**) Control sampling site at the mouth of the Simeto river (37°24′59′′ N, 15°05′16′′ E), blue placeholder. Both images were modified from Google Earth.

The sampling areas were chosen to obtain specimens both from a site with a high anthropic impact (Priolo Gargallo, a site of national interest included among the SINs in art. 1, paragraph 4 of the Law of December 9, 1998, n. 426 “New interventions in the environmental field”) and from a control site represented by a protected area (Oasi del Simeto) (Figure 1).

A total of 20 specimens were collected at the two sites (N = 10, Oasi del Simeto; N = 10, Priolo Gargallo) during May and June 2019 and 2022 and stored in 96% ethanol before continuing with the experimental analysis. Extraction of total DNA was performed using the DNeasy Blood and Tissue Kit (Qiagen, Hilden, Germany), following the manufacturer’s protocol with some modifications. Before proceeding with the next steps, the DNA concentration in each extracted sample was quantified using the Nanodrop ND-1000 spectrophotometer (Thermo Fisher Scientific, Waltham, MA, USA): the absorbance levels at 230, 260, and 280 nm and the respective ratios were measured because they allowed evaluation of the quality of the extract.

### 2.2. DNA Barcoding

The *COI* gene was amplified in a subset of *O. heydeni* specimens using the PCR conditions reported in [25]: an initial denaturation step at 94 °C for 15 min, followed by 35 cycles (denaturation at 94 °C for 30 s, annealing at 51 °C for 1 min, extension at 72 °C for 1 min), and a final extension step at 72 °C for 10 min. The reaction was executed in a final volume of 50 µL containing the primers described in [26]. Negative controls were included in all PCR runs to check for cross-contamination. The obtained amplicons were verified by electrophoresis on a 1% agarose gel and displayed through a Safe Imager TM 2.0 Blue Light Transilluminator (Thermo Fisher, Waltham, MA, USA) using the SYBR^®^ Safe dye (Thermo Fisher Scientific, Waltham, MA, USA). The QIAquick PCR purification kit (Qiagen, Hilden, Germany) was used to purify all amplicons, which were then bidirectionally sequenced by EUROFINS GENOMICS S.r.l. (Milan, Italy). Species identification was performed by analysis of similarity with the Basic Local Alignment Search Tool (BLAST), megablast algorithm, against the GenBank nucleotide database (https://www.ncbi.nlm.nih.gov (accessed on 15 March 2023), and the pairwise distance (p-distance) was calculated using the MEGA X software [27]. All sequences were submitted to the GenBank database.

### 2.3. mtDNAcn

The relative mtDNAcn was determined by qPCR through the comparative Ct method [28], using the mt *COI* mini barcode as the target gene and the nuclear *18S* gene as the reference gene. All samples were run in three replicates on a QuantStudio 1 Real-Time PCR System (Thermo Fisher Scientific, Waltham, MA, USA) using the following primer pairs: miniCOI-F 5′–GCACCAGACATAGCATTCCC–3′ and miniCOI-R 5′–ATACAGTTCAACCCGTCCC–3′ designed using Primer3 and 18S-FW 5′–TGTGCCGCTAGAGGTGAAATT–3′ and 18S-REV 5′–GCAAATGCTTTCGCTTTCG–3′ [29].

Each qPCR reaction was performed in a final volume of 20 µL using SensiFAST SYBR master mix (Meridian BIOSCIENCE, Cincinnati, OH, USA) with ROX reference dye low concentration (INVITROGEN) following manufacturer instructions, and a negative control was included for each qPCR assay. A specific thermal profile was then established: 95 °C for 3 min, followed by 40 cycles of 95 °C for 20 s and 60 °C for 20 s. A final step of 95 °C for 15 s, 60 °C for 1 min, and 95 °C for 1 s was included.

Cycle threshold (Ct) values of mitochondrial PCR products were normalized to Ct-values of the nuclear locus according to the equation:

*relative mtDNAcn* = 2 × 2^ΔCt^

where ΔCt = Ct (nDNA gene) − Ct (mtDNA gene), i.e., the difference between the Ct of the nuclear gene and the Ct of the mitochondrial gene, was calculated [28].

All data were subjected to statistical analysis using GraphPad Prism software version 8.3.0. The Mann-Whitney test was conducted to verify the statistical significance of the mtDNAcn variation between the sampling sites, accepting as significant values those with a *p*-value < 0.05.

## 3. Results

### 3.1. DNA Barcoding

A 654 bp-long portion of the *COI* gene was amplified from each sample. The obtained sequences were, for the first time, linked to morphologically identified specimens. No insertions, deletions, or stop codons were observed. The lack of stop codons and the length of the amplified sequences suggest that they are functional mitochondrial *COI* sequences. The sequences used as query sequences for BLAST search yielded a percentage of identity, with their top-match sequences ranging between 83.21% and 84.12% with a coverage percentage of 100%. No *O. heydeni* sequences were found in the database, and the congeneric species to which our sequences align is *Opsius stactogalus* (Table 1). The average p-distance calculated in MEGA X between the two species was 21.5%, while the average intraspecific p-distance of *O. heydeni* was 0.65%.

### 3.2. mtDNAcn Variation

**Figure 2:**
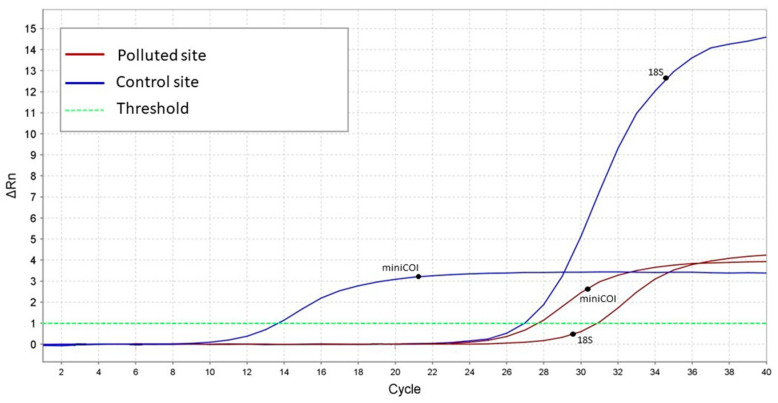
An example of a qPCR result comparing one sample from each site (polluted vs. control) to display how relative mtDNAcn was determined. The amplification plot shows the rate of fluorescence of the SYBR green dye, indicated as ΔRn, vs. the number of PCR cycles: a lower cycle threshold (Ct) represents more DNA copies. The amplification plot shows a lower biomarker level for the polluted site specimen (red curve) due to greater mitochondrial damage.

**Figure 3:**
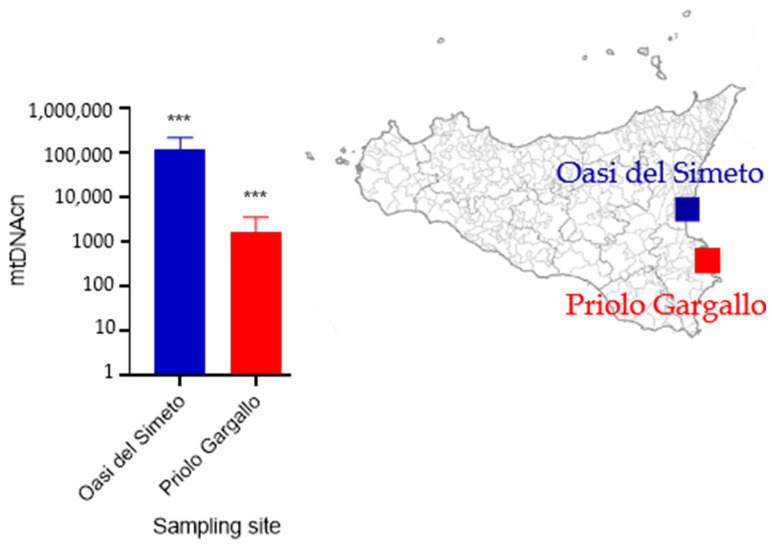
Relative mtDNAcn measured in *O. heydeni* specimens from Oasi del Simeto and Priolo Gargallo. The Mann-Whitney test resulted in a *p*-value < 0.001 (***).

The mtDNAcn was lower on the polluted site (Priolo Gargallo) compared to the control site (Oasis of the Simeto) (Supplementary Materials Table S1). As reported in Figure 2, the cycle threshold (Ct), representing the number of PCR cycles required before the fluorescence level of each amplified product reached the threshold level, was lower in targets with a higher amplification rate. In particular, while the Ct values for the nuclear target were similar for the two sites, the Ct values for the mitochondrial target were very different, with a higher value for the polluted site. To assess the presence of specific qPCR products, the amplicons were also visualized using a 2% electrophoretic agarose gel. In addition, the amplification plot displays a lower ΔCt for the template of the contaminated area (Priolo Gargallo) compared to that of the control site (Oasis of Simeto), indicating a decreased mtDNA level in the former (Figure 2 and Figure 3).

In fact, the specimens from the polluted area had a mean of 1.116 mtDNA copies per cell, while the control population had a mean of 119.395 mtDNA copies per cell, indicating a reduction in the first population of more than 99%. The Mann-Whitney test revealed a statistically significant difference in the mtDNAcn means between the two populations (*p*-value < 0.001).

## 4. Discussion

Two main objectives were achieved in the present study: (i) the *COI* barcode sequences of *O. heydeni* were obtained for the first time, and (ii) a statistically significant difference in the number of copies of the mitochondrial genome was detected in two populations of this species living at the Priolo Gargallo site, affected by high anthropic impact and the presence of contaminants, and at the control site Oasi del Simeto. The choice to investigate *O. heydeni* derives from the particular ecology of this species, which is a sap-feeder on *Tamarix* spp., plants that are able to live on polluted sites where they bioaccumulate high levels of heavy metals in the bark and leaves. For example, a significant positive correlation was found between metal concentrations in soil and the leaf and bark of *T. aphylla* in Tunisia [24]. The Magnisi peninsula at the Priolo Gargallo site shows a high degree of Pb and Hg contamination [19] and hosts sparse vegetation of *Tamarix* grown on a pyrite ash dump. Therefore, we speculate that the polluted soil on which *Tamarix* plants grow, coupled with the high bioaccumulation capacity of these plants, on whose sap *O. heydeni* feeds, affected the mtDNAcn of this species of insect.

As regards the *COI* sequences of *O. heydeni*, the BLAST search yielded no match; however, our sequences align with those of *O. stactogalus* with a low percentage of identity, ranging between 83.21% and 84.12%, with 100% sequence coverage. *O. stactogalus* shares the general appearance, dimensions, and ecology of *O. heydeni*, although the two species are quite distinct based on the morphology of the male genital apparatus. Therefore, in this study, the *COI* barcode sequences were linked for the first time with specimens of *O. heydeni* that were identified on the basis of their morphology.

The results of the mtDNAcn analyses demonstrate that the number of copies of mitochondrial DNA decreased significantly in the population of the polluted site compared with the control site. Similar results have been obtained in other species of invertebrates under different starvation and environmental pressures [30,31].

In agreement with our results, a recent study [32] showed a decrease in the mtDNAcn as a result of pesticide exposure in *Bombus terrestris,* one of the most important pollinators in agriculture, which is currently threatened with extinction mainly due to intensive use of xenobiotics. It is interesting to note that in the same study, the authors demonstrated that different pesticides, having different molecular targets, led to increases in the biomarker as a compensatory mechanism for the inhibition of mitochondrial respiration [32]. These observations highlight how the same biomarker might show different responses depending on the mechanisms of drug action, the drug’s chemical nature, and the transfer pathways.

Furthermore, in certain circumstances, depending on changes in environmental effects, either an increase or a decrease in the number of mitochondrial copies can be observed. For example, in a study on *Palaemon carinicauda*, a crustacean of significant economic interest in Chinese aquaculture, the mtDNAcn increased with rising temperatures, whereas rising salinity resulted in an initial increase in the same biomarker followed by a decrease. In the first case, the increased mtDNAcn was probably due to the fact that higher temperatures can increase the rate of energy metabolism, thus suggesting the continued replication of mtDNA (and hence an increase in its copy number) to meet metabolic requirements. On the other hand, it is known that during osmotic pressure regulation, due to water salinity variation, ROS are generated, which induces oxidative stress. It is, therefore, possible that above a threshold of increased salinity values (and the simultaneous stimulation of mtDNA replication and ROS accumulation), a polymerase block occurred and, consequently, the copy number decreased [31].

In *Drosophila melanogaster*, a species of insect used as a model organism, there is an overall reduction in the number of copies of the mitochondrial genome with increasing age; in this case, the older specimens are unable to deal with stress factors such as temperature changes or starvation without damage at the mitochondrial level [30].

The mechanisms by which environmental pollutants affect mtDNAcn variation are not fully known. However, the variation of mtDNAcn should be interpreted in light of the mitochondrial dynamics triggered by environmental toxicants and involving mitochondrial fission and fusion as regulatory factors of mtDNAcn [33]. Currently, mtDNAcn variation as a biomarker in ecotoxicological studies to assess environmental health status is underutilized since most research focuses on human implementation. Variation in the ratio of mitochondrial to nuclear genomes is considered a biomarker of mitochondrial dysfunction for many human pathologies [34], related to exposure to various environmental contaminants such as lead, PAH, benzene, particulate matter, and arsenic [35,36,37,38,39]. Conversely, our study adds information on the response of the mtDNA to stress factors in wildlife organisms.

## 5. Conclusions

The present study highlights the statistically significant decreases in the mtDNA copy number in insects living in the polluted area of Priolo Gargallo compared to those living in the control site. Although we have not evaluated the level of pollutant bioaccumulation in *Opsius heydeni*, we demonstrated, supported also by the scientific literature, how the mtDNAcn undergoes significant variations probably because of environmental stressors. These preliminary findings indicate that the mtDNAcn could be used as a biomarker of exposure to assess environmental quality, allowing early detection of genomic damage in animals caused by pollutants. Furthermore, these data confirm that Hemiptera are good ecological indicators of environmental changes and could be used to monitor a specific terrestrial ecosystem and its stress level [40] and suggest that *O. heydeni* is a good candidate as a bioindicator species to study environmental quality and conditions.

Finally, from a future perspective, the early assessment of mtDNA alterations caused by pollutants should be the subject of more intensive research in the fields of health and environmental toxicology.

## Figures and Tables

**Table 1:** COI DNA Barcode sequences of *O. heydeni*.

Sample Code	GenBank	Species Matched by BLAST	Matched GenBank	% Identity
	Accession N°		Accession from BLAST	100% Coverage
Oh1	OQ661886	*Opsius stactogalus*	HQ929190	83.66
Oh2	OQ661887	*Opsius stactogalus*	HQ929190	84.12
Oh3	OQ661888	*Opsius stactogalus*	HQ929190	83.97
Oh4	OQ661889	*Opsius stactogalus*	HQ929190	83.51
Oh5	OQ661890	*Opsius stactogalus*	HQ929190	83.66
Oh6	OQ661891	*Opsius stactogalus*	HQ929190	83.21

## Data Availability

The data presented in this study are available on request from the corresponding authors.

## Supplementary Materials

The following supporting information can be downloaded at: https://www.mdpi.com/article/10.3390/ani13111793/s1. Table S1: Raw data of DNA sample concentrations and relative mtDNAcn.
